# Dysregulation of Leukaemia Inhibitory Factor (LIF) Signalling Pathway by Supraphysiological Dose of Testosterone in Female Sprague Dawley Rats During Development of Endometrial Receptivity

**DOI:** 10.3390/biomedicines13020289

**Published:** 2025-01-24

**Authors:** Allia Najmie Muhammad Yusuf, Mohd Fariz Amri, Azizah Ugusman, Adila A Hamid, Izzat Zulhilmi Abd Rahman, Mohd Helmy Mokhtar

**Affiliations:** 1 Department of Physiology, Faculty of Medicine, Universiti Kebangsaan Malaysia, Cheras, Kuala Lumpur 56000, Malaysia; allia.najmie@ums.edu.my (A.N.M.Y.); dr.azizah@ukm.edu.my (A.U.); adilahamid@ukm.edu.my (A.A.H.); izzat_zulhilmi@ukm.edu.my (I.Z.A.R.); 2 Department of Biomedical Sciences, Faculty of Medicine and Health Sciences, Universiti Malaysia Sabah, Kota Kinabalu 88400, Malaysia; 3 Department of Pathology and Microbiology, Faculty of Medicine and Health Sciences, Universiti Malaysia Sabah, Kota Kinabalu 88400, Malaysia; fariz@ums.edu.my

**Keywords:** testosterone, hyperandrogenism, endometrial receptivity, window of implantation, LIF, JAK1, STAT3

## Abstract

Objective: This study investigated the effects of a supraphysiological dose of testosterone on uterine morphology and the regulation of the leukaemia inhibitory factor (LIF) signalling pathway during endometrial receptivity. Methods: In this study, 30 adult female Sprague–Dawley rats were divided into treatment and control groups. The treatment groups received subcutaneous injections of 1 mg/kg/day of testosterone from gestational day 1 to day 3, either testosterone alone or in combination with inhibitors (anastrozole, finasteride, or both). A control group of six untreated rats was maintained for comparison. Rats were euthanised on the evening of gestational day 4 to examine uterine morphological changes, gene expression and the distribution of proteins associated with the LIF signalling pathway (LIF, LIFR, JAK1 and STAT3) and MUC1 by quantitative polymerase chain reaction (qPCR) and immunohistochemistry (IHC), respectively. Results: The results of this study showed that the thickness of the endometrium and myometrium, as well as the number of glands, markedly decreased in all testosterone-treated rats. In addition, the mRNA levels of *LIF*, *LIFR*, *JAK1* and *STAT3* were significantly downregulated in response to supraphysiological testosterone treatment, while the mRNA of *MUC1* was significantly upregulated. The IHC results were consistent with the mRNA data and confirmed the changes in protein distribution in all treatment groups. Conclusions: A supraphysiological dose of testosterone may impair endometrial receptivity through dysregulation of the LIF signalling pathway, potentially affecting fertility.

## 1. Introduction

Endometrial receptivity is a critical period during which the endometrium is optimally prepared for embryo implantation. This transient state is also known as the implantation window and lasts approximately two to four days in humans, typically around days 19 to 23 of a 28-day menstrual cycle [1]. The regulation of endometrial receptivity involves the integration of hormonal signalling, including oestrogen, primarily oestradiol (E2) and progesterone, as well as local factors including growth factors and cytokines [2,3]. These molecules coordinate endometrial remodelling by influencing the expression of adhesion molecules, immunomodulators and enzymes responsible for extracellular matrix (ECM) degradation. The orchestration of these processes is essential for achieving the dynamic equilibrium required for embryo implantation [4]. The dysregulation of endometrial receptivity, caused by hormonal imbalance or altered signalling pathways, can significantly impair the implantation potential and is a key factor in reproductive disorders such as recurrent implantation failure and infertility [5].

The endometrium consists of three functional layers, namely the luminal epithelium, the glandular epithelium and the stroma, each of which plays a decisive role in the implantation of the embryo. The luminal epithelium, a monolayer of columnar cells, acts as an initial barrier to the blastocyst and contains adhesion molecules, including integrins and selectins, which promote attachment. During the implantation window, anti-adhesive molecules such as mucin-1 (MUC1) are downregulated to promote embryo adhesion [6]. The glandular epithelium, lining the endometrial glands, secretes nutrients, cytokines and growth factors such as leukaemia inhibitory factor (LIF) that support embryonic development and communication with the endometrium [7]. Meanwhile, the stroma, which consists of fibroblasts, immune cells and the extracellular matrix, is decidualised and transforms into decidual cells that provide mechanical support and secrete factors important for implantation and placental development [4]. The thickness of the endometrium, typically over 7 mm, with a balanced ratio between the epithelial and stromal layers, is also critical for receptivity [8,9]. However, an abnormally thickened endometrium can impair receptivity, and this dysfunction can lead to delayed maturation, increased secretion of anti-adhesive molecules and impaired embryo implantation [10].

In addition, the LIF signalling system plays a crucial role in the regulation of endometrial receptivity and embryo implantation. LIF is a cytokine that is primarily produced by the glandular epithelium and is considered essential for successful implantation. LIF exerts its effect by binding to its receptor complex, which consists of the leukaemia inhibitory factor receptor (LIFR) and gp130 and is present on the surface of the luminal and glandular epithelial cells. This binding activates downstream signalling cascades involving Janus kinase 1 (JAK1) and the signal transducer and activator of transcription 3 (STAT3) [11,12]. STAT3, a downstream target of LIF, is phosphorylated in the luminal epithelium prior to embryo implantation [11]. The inhibition or conditional deletion of STAT3 in utero leads to the failure of implantation of mouse embryos [4,13]. Following its activation, JAK1 phosphorylates STAT3, leading to its dimerisation and subsequent translocation to the nucleus. There, it modulates the expression of genes that are crucial for implantation, such as genes involved in cell proliferation, immune tolerance and the regulation of adhesion molecules [12]. STAT3 plays a crucial role in suppressing the production of MUC1, a mucin glycoprotein that forms an anti-adhesive barrier on the luminal epithelium. By reducing MUC1 expression, the LIF-STAT3 signalling pathway facilitates the adhesion of the embryo to the endometrium [11,12]. Therefore, disturbances of the LIF signalling pathway due to hormonal imbalances or genetic mutations can severely impair endometrial receptivity and lead to implantation failure [14].

Testosterone in supraphysiological doses has a negative effect on endometrial receptivity and embryo implantation, as is the case with hyperandrogenic disorders such as polycystic ovary syndrome (PCOS) [15,16]. Hyperandrogenism, which is characterised by elevated testosterone levels, may disrupt endometrial receptivity and embryo implantation by interfering with the action of E2 and progesterone, which are essential for endometrial preparation [17]. Excess testosterone has been linked to altered cytokine signalling and adhesion molecule expression, resulting in a non-receptive endometrial environment [2]. However, the effects of testosterone at supraphysiological doses on the LIF signalling pathway during endometrial receptivity have not yet been elucidated. This study therefore investigated the effects of a supraphysiological dose of testosterone on uterine morphology and the regulation of the LIF signalling pathway during the receptive state of the endometrium. Understanding the effects of elevated testosterone on the endometrium in relation to the LIF signalling pathway is crucial for the development of strategies to improve fertility under hyperandrogenic conditions.

## 2. Materials and Methods

### 2.1. Animal Preparation and Hormonal Treatment

Thirty adult female Sprague–Dawley rats (n = 6 per group) weighing 225 ± 25 g with at least two consecutive normal oestrous cycles were obtained from the Laboratory Animal Resources Unit (LARU), Faculty of Medicine, Universiti Kebangsaan Malaysia. The rats were housed individually in a controlled environment at a temperature of 24 ± 2 °C with a 12 h light/dark cycle (lights on from 06:00 to 18:00). They received a normal diet from Envigo (Inotiv, IN, USA) and water ad libitum. The Animal Ethics Committee of the Universiti Kebangsaan Malaysia approved all experimental procedures (approval number: FISIO/PP/2019/MOHD HELMY/30-OCT./1060-OCT.-2019-AUG.-2022).

**Figure 1:**
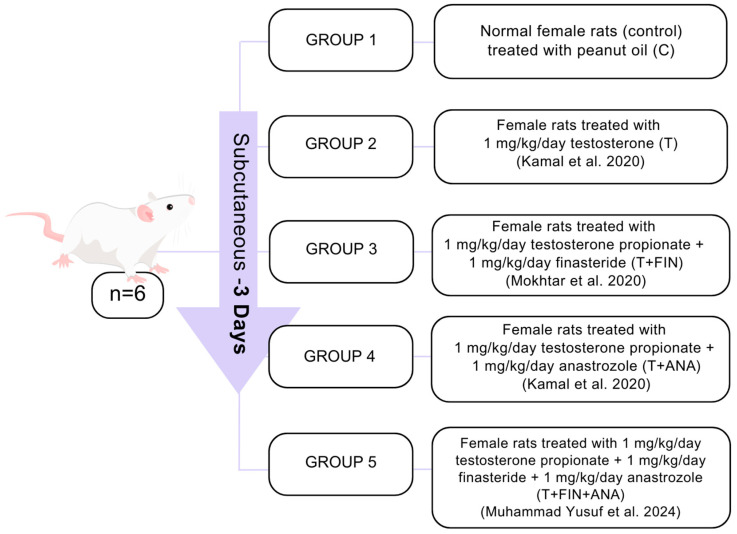
A diagram showing the grouping and treatment of the animals [16,20,21].

During the proestrus phase, each female rat was mated overnight at a 1:1 ratio with a male of the same species. Successful mating was confirmed the next morning by the detection of sperm in vaginal swabs or the presence of a vaginal plug (referred to as postcoital day 1) [18,19]. Rats were subsequently allocated randomly to one of five groups (n = 6 per group), as shown in Figure 1 below.

Treatments were administered subcutaneously behind the scruff of the neck on three consecutive days, from the first to the third day of gestation (early gestation phase) [20]. The substances, specifically testosterone propionate, finasteride and anastrozole (Sigma-Aldrich, St Louis, MO, USA), were solubilized in 0.1 mL of peanut oil before administration. Finasteride and anastrozole were administered 30 min before testosterone propionate [16,22]. The high dose of testosterone propionate was selected based on its use in previous studies [21].

On the evening of the fourth day of gestation, which coincides with the endometrial receptivity phase in rats [22], the animals were euthanised by intravenous administration of a high-dose ketamine–xylazine mixture (0.3 mL per 100 g body weight). Uterine tissue was subsequently collected for further analyses focusing on the gene expression and protein distribution of LIF, LIFR, STAT3, JAK1 and MUC1, as well as morphological changes in the uterus.

### 2.2. Histomorphological Analysis of the Uterus

After the rats were sacrificed, the uterus was excised and the surrounding fatty tissue was removed. The specimen was then stored in a 10% neutral buffered formalin solution. One-third of the uterus was then chemically processed and embedded in paraffin. The uterine tissue was then sectioned into 5 μm thick slices utilising a Leica RM2245 microtome (Leica Biosystems, Wetzlar, Germany), air-dried, deparaffinised, successively rehydrated and stained with haematoxylin and eosin (H&E) solution. Slides were then examined using an Olympus BX40 light microscope (Olympus Corporation, Tokyo, Japan). Finally, the images were evaluated by two individuals (an anatomic pathologist and a PhD candidate). In brief, histomorphological parameters such as the thickness of the endometrium, myometrium and epithelial cell height, as well as cellular details such as the presence of glandular structures, stromal density and the condition of the epithelial lining, were assessed. The data obtained from the histomorphological examination were statistically analysed to determine the significance of the differences between the control and treated groups.

### 2.3. Protein Distribution Analysis by Immunohistochemistry (IHC)

The uteri were immersed overnight in a neutral, buffered formaldehyde solution for fixation. They were then treated with a higher-concentration ethanol solution to remove the water. Before the samples were made into sections with a thickness of 5 mm, the uteri underwent a chemical process and were encased in paraffin wax. The slides were then treated with Dako’s antigen retrieval solution (Glostrup, Denmark) at pH 6.0 and pH 9.0. The slides were treated with hydrogen peroxide to inhibit the naturally occurring peroxidase. Samples were then treated with the Mouse- and Rabbit-Specific HRP/DAB IHC Detection Kit—micro-polymer blocking serum (Cat ab236466, Abcam, Cambridge, MA, USA). To examine the distribution of the LIF signalling proteins, the slides were treated with a rabbit polyclonal antibody targeting the leukaemia inhibitory factor receptor (LIFR) at a dilution of 1:250 (Cat. AB101228 Abcam), or a rabbit antibody against the signal transducer and activator of transcription 3 (STAT3) at a dilution of 1:1500 (Cat. AB119352 Abcam). In addition, a rabbit antibody targeting Janus kinase-1 (JAK1) at a dilution of 1:100 (Cat. AB125051 Abcam) and a rabbit antibody against mucin-1 (MUC1) at a dilution of 1:250 (Cat. AB109185 Abcam) were utilized. The slides were subsequently incubated with the micropolymeric secondary antibody from the Mouse- and Rabbit-Specific HRP/DAB IHC Detection Kit (Cat ab236466, Abcam). Furthermore, the use of the DAB substrate from the Mouse and Rabbit Specific HRP/DAB IHC Detection Kit-Micro-polymer (catalogue number ab236466, Abcam) facilitated the identification of the protein. Finally, the slides were stained with haematoxylin and then gradually dehydrated. The slides were analysed using an Olympus BX53 light microscope (Olympus Corporation, Tokyo, Japan). An Olympus DP27 (Olympus Corporation, Tokyo, Japan) colour camera system was used to examine and photograph the stained samples. For all experimental groups, all pictures were taken in the same settings. The immunostaining analyses were conducted utilizing the established semi-quantitative Quickscore methodology, offering an intensity score ranging from 0 to 3 and a ratio score from 0 to 5. Scores of 0 and 2 are considered negative while scores ranging from 3 to 8 are considered positive [23,24,25]. The analysis was performed by two observers, one of whom was an anatomical pathologist and the other a PhD student. At least one of the observers was unaware of the categorisation of the group being studied. In cases of disagreement, the analyses were evaluated jointly, and an agreement was attained. Table 1 shows the specifics of the QS scoring.

### 2.4. Analysis of mRNA Expression Using Quantitative Polymerase Chain Reaction (qPCR)

To preserve the RNA in the cells and ensure its integrity, the rat uteri were fixed in an RNA-Later solution (Sigma Aldrich, Saint Louis, MO, USA). RNA was then isolated from the uterus using the Nucleospin RNA isolation kit (CAT 740955.50, Macherey-Nagel, Duren, Germany) according to the manufacturer’s instructions. To assess RNA purity, samples were analysed for absorbance at 260 nm and 280 nm, utilizing the 260/280 ratio (Gene Quant 1300, Cambridge, UK). Then, 400 ng of RNA was converted to complementary DNA (cDNA) utilising the qPCRBio cDNA synthesis kit (CAT PB30.11-10, PCR Biosystems, London, UK). As a control, this material was amplified without the addition of reverse transcriptase (-RT). The qPCR master mix was prepared using the qPCRBio SyGreen Blue Mix Kit (CAT PB20.17-05, PCR Biosystems). One microliter of cDNA was used along with the *glyceraldehyde-3-phosphate dehydrogenase (GAPDH),* which is used as a reference gene in this study. The specific primers for the following genes were used for qPCR analysis including the *leukaemia inhibitory factor receptor (LIFR)* (catalogue number: RQP050963, GeneCopoeia, Rockville, MD, USA), the *leukaemia inhibitory factor (LIF)* (catalogue number: RQP050332, GeneCopoeia), *Janus kinase 1 (JAK1)* (catalogue number: RQP067291, GeneCopoeia), *signal transducer and activator of transcription 3 (STAT3)* (catalogue number: RQP049074, GeneCopoeia), *mucin-1 (MUC1)* (catalogue number: CS-RQP2143L-01, GeneCopoeia) and *GAPDH* (catalogue number: RQP050332, GeneCopoeia). Real-time PCR was conducted utilising the BioRad CFX96 Real-Time System under the specified settings: polymerase activation at 95 °C for 5 min, denaturation at 95 °C for 10 s (repeated for 40 cycles) and annealing and elongation at 60 °C for 30 s (also repeated for 40 cycles). The experiment was performed three times each. Finally, data were analysed using the comparative CT approach (2^−∆∆Ct^). In this approach, the normalised quantity of each gene was compared to the normalised quantity of the reference gene to determine the relative quantity of each amplicon.

### 2.5. Statistical Analysis

In this study, GraphPad Prism version 10.2.3 was used for statistical analysis. The Shapiro–Wilk normality test was applied to assess the normality of the data. The data were analysed using parametric analysis of variance (ANOVA) followed by a post hoc test using the Dunn test or Kruskal–Wallis test if the data distribution was non-normal. The difference was considered statistically significant if *p* < 0.05.

## 3. Results

### 3.1. Effects of High Dose of Testosterone on the Morphological Changes in the Uterus

**Figure 2:**
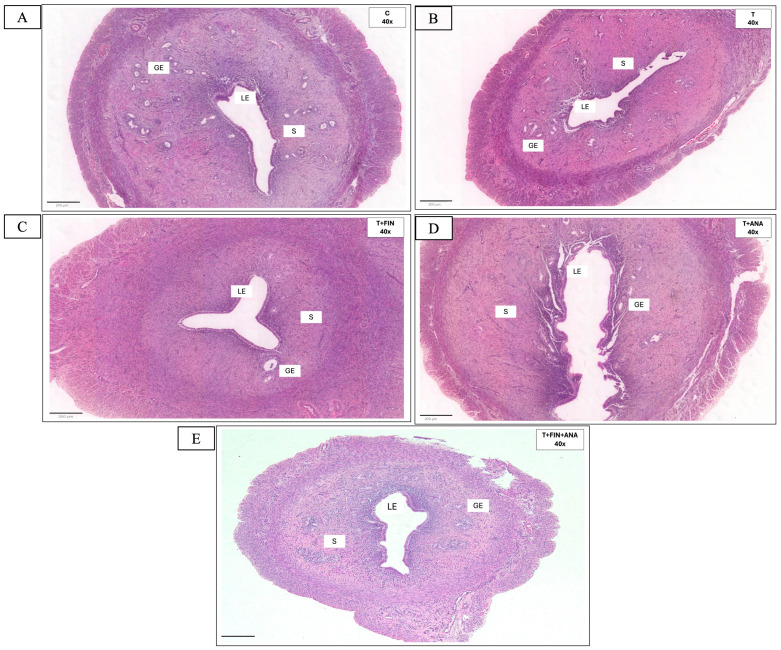

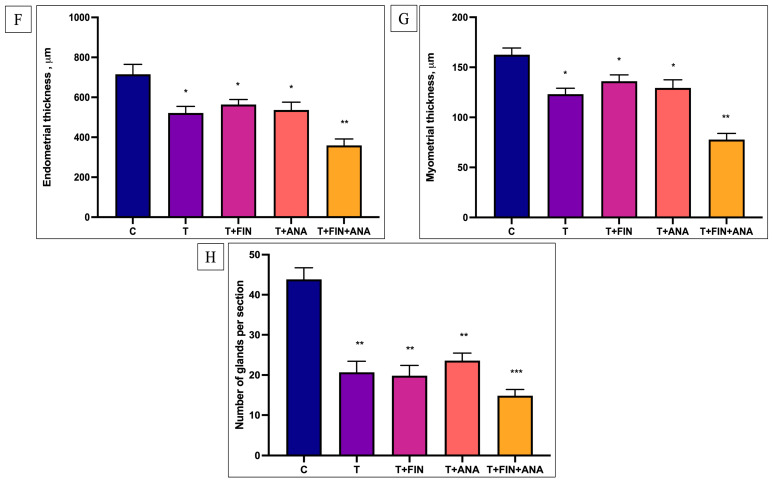
H&E staining of rat uterus tissue is shown; (**A**) H&E ×40 magnification from rat treated with vehicle (C); (**B**) H&E ×40 magnification from rat treated with testosterone propionate (T); (**C**) H&E ×40 magnification from rat treated with testosterone + finasteride (T+FIN); (**D**) H&E ×40 magnification from rat treated with testosterone + anastrozole (T+ANA); and (**E**) H&E ×40 magnification from rat treated with testosterone + finasteride + anastrozole (T+FIN+ANA). (**F**–**H**) Graphs show endometrial and myometrial thickness and average number of glands per section. GE, glandular epithelium; LE, luminal epithelium, S, stroma. Scale bar = 1 mm, scale bar = 200 μm, magnification 40× and 200×. Error bars denote the standard error of the mean (SEM). n = 6 in each group, * *p* < 0.05, ** *p* < 0.01, *** *p* < 0.001 indicate significance relative to the normal control group.

Figure 2 shows the uterine sections of each group and the effects of the different treatments on the uterine tissue, particularly the thickness of the endometrium, the thickness of the myometrium and the number of glands per section for each group. Each graph is analysed in comparison with the control group and the different treatment groups.

The control group has the highest endometrial thickness, with a mean thickness of 800 ± 49.3 μm. The testosterone propionate (T), testosterone + finasteride (T+FIN) and testosterone + anastrozole (T+ANA) treatment groups showed significantly lower mean endometrial thickness (*p* < 0.05), with the testosterone + both inhibitors (T+FIN+ANA) group significantly more affected (*p* < 0.01) as compared to the control group. There was no significant difference between the T, T+FIN and T+ANA groups. Treatment with testosterone alone significantly reduced the thickness of the endometrium to almost 500 ± 33 μm (*p* < 0.05). Meanwhile, testosterone in combination with finasteride showed a similarly significant reduction in thickness (*p* < 0.05) compared to the control group, with the value being similar to that of the testosterone-only group. The combination of testosterone with anastrozole also maintained this reduced thickness (*p* < 0.05), suggesting that finasteride and anastrozole do not appear to counterbalance the effect of testosterone on endometrial thickness. The combination of testosterone with finasteride and anastrozole demonstrates a remarkable reduction (*p* < 0.01), suggesting a greater effect of the combination of both blockers, with endometrial thickness decreasing to approximately 400 ± 32.4 μm.

Similarly, the control group exhibited the highest myometrial thickness at 160 ± 6.6 μm, while the testosterone propionate (T), testosterone + finasteride (T+FIN) and testosterone + anastrozole (T+ANA) treatment groups showed significantly reduced mean myometrial thickness (*p* < 0.05), with the testosterone and both inhibitors group (T+FIN+ANA) being significantly more affected compared to the control group (*p* < 0.01). There was no significant difference between the T, T+FIN and T+ANA groups. The combination of testosterone, finasteride and anastrozole showed the lowest mean myometrial thickness with a reduction to about 80 ± 6.2 μm.

The testosterone treatment also influences the number of endometrial glands. The average number of glands per section is highest in control groups at about 45 ± 2.9. There was no significant difference between the T, T+FIN and T+ANA groups. The combination of testosterone with finasteride and anastrozole showed the most significant reduction (*p* < 0.001), with the number of glands decreasing to about 15 ± 1.6 per section, indicating an additive or synergistic effect when both inhibitors are combined with testosterone.

### 3.2. Protein Distribution and mRNA Expression of Leukaemia Inhibitory Factor (LIF) and Leukaemia Inhibitory Factor Receptor (LIFR)

**Figure 3:**
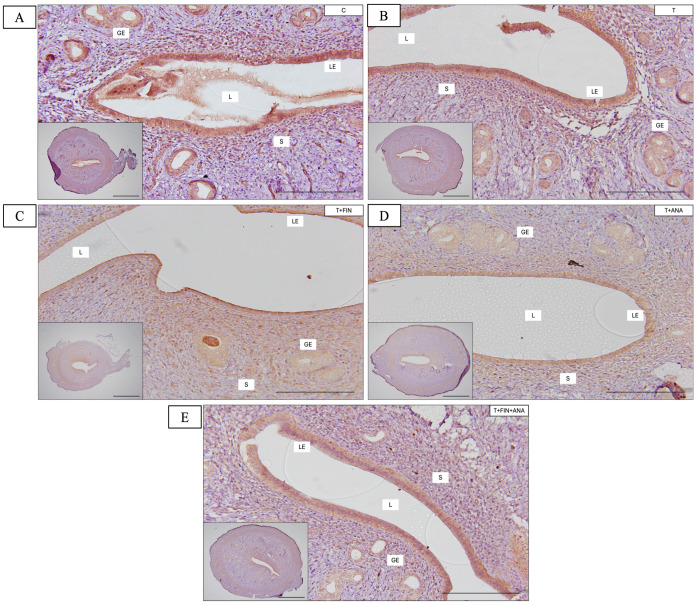

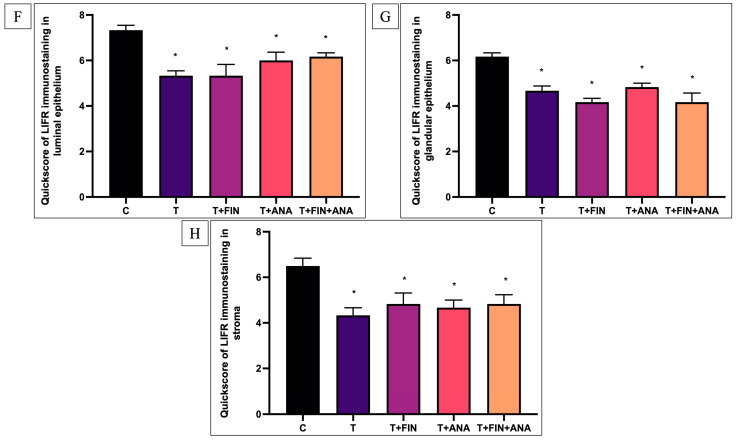
Distribution of leukaemia inhibitory receptor (LIFR) in the uterus of rats. (**A**–**E**) The antibody binding site of LIFR, which can be seen in the stroma and luminal and glandular epithelium of the uterus, is coloured brown. Compared to the control group, all four treatment groups (T, T+FIN, T+ANA and T+FIN+ANA) showed a lighter brown discolouration in all compartments. (**F**–**H**) Semi-quantitative assessment of LIFR immunostaining for the endometrial compartments. LE: Luminal epithelium; GE: Glandular epithelium; L: Lumen of the endometrium; S: Stroma. Scale bar = 200 μm, scale bar insert = 1 mm, magnification 40× and 200×. Error bars represent standard error of the mean (SEM). n = 6 in each group, * *p* < 0.05 indicates significance relative to the normal control group. C: control; T: testosterone propionate; T+FIN: testosterone propionate + finasteride; T+ANA: testosterone propionate + anastrozole; T+FIN+ANA: testosterone propionate + finasteride + anastrozole.

Figure 3 depicts the distribution and Quickscore of LIFR across various uterine compartments in rats subjected to different treatments. In the first graph, the control group shows the highest LIFR immunostaining score in the luminal epithelium. The group treated with testosterone shows a significant decrease in LIFR immunostaining compared to the control group, with a Quickscore of about 5 (*p* < 0.05). The combination of anastrozole or finasteride with testosterone demonstrated a significant reduction in immunostaining relative to the control group (*p* < 0.05). A similar pattern was observed when both inhibitors were combined with testosterone, resulting in a significant decrease in LIFR immunostaining compared to the control group (*p* < 0.05).

Looking at the glandular epithelium, the control group shows the highest LIFR immunostaining activity. The testosterone-treated group exhibits a substantial reduction in LIFR expression relative to the control group (*p* < 0.05). A similar pattern can be observed in the other treatment groups with the inhibitors, where LIFR immunostaining is significantly reduced compared to the control group (*p* < 0.05). The trend is also similar in the stromal compartment, where the control group exhibits elevated LIFR immunostaining activity in the stroma. The expression of LIFR has substantially decreased in the treatment with the testosterone group relative to the control group. Treatment of testosterone with finasteride or anastrozole or a combination of both inhibitors also showed a substantial decrease in LIFR immunostaining, similar to the testosterone-only group (*p* < 0.05).

**Figure 4:**
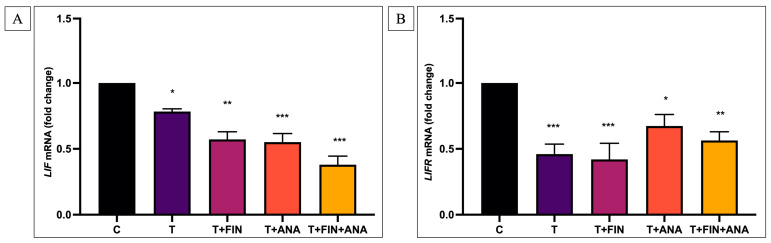
Effects of testosterone on (**A**) *LIF* and (**B**) *LIFR* mRNA expression. n = 6 in each group, error bars represent standard error of the mean (SEM). * *p* < 0.05, ** *p* < 0.01, *** *p* < 0.001 indicate significance relative to the normal control group. C: normal control; T: testosterone propionate; T+FIN: testosterone propionate + finasteride; T+ANA: testosterone propionate + anastrozole; T+FIN+ANA: testosterone propionate + finasteride + anastrozole.

Figure 4A illustrates the expression of *LIF* mRNA. This study demonstrates that testosterone administration significantly downregulates *LIF* mRNA expression (*p* < 0.05) relative to the control groups. The additional administration of finasteride led to a further downregulation of *LIF* mRNA expression relative to the control group (*p* < 0.01). *LIF* mRNA expression was considerably downregulated in the anastrozole treatment group relative to the control group (*p* < 0.001). A comparable pattern was observed in the group receiving the combination of testosterone with both inhibitors, demonstrating downregulation of *LIF* mRNA expression while retaining statistical significance (*p* < 0.001) relative to the control group.

Figure 4B illustrates the expression of *LIFR* mRNA. This investigation demonstrates the treatment with testosterone alone and in conjunction with finasteride results in a significant downregulation of *LIFR* mRNA expression (*p* < 0.001) relative to the control groups. Meanwhile, the additional administration of anastrozole showed significant downregulation of *LIFR* expression relative to the control group with statistical significance (*p* < 0.05). However, the combined treatment of testosterone with both inhibitors resulted in a further downregulation of *LIFR* mRNA expression compared to the control group with the same statistical significance (*p* < 0.01).

### 3.3. Protein Distribution and mRNA Expression of Janus Kinase 1 (JAK1)

**Figure 5:**
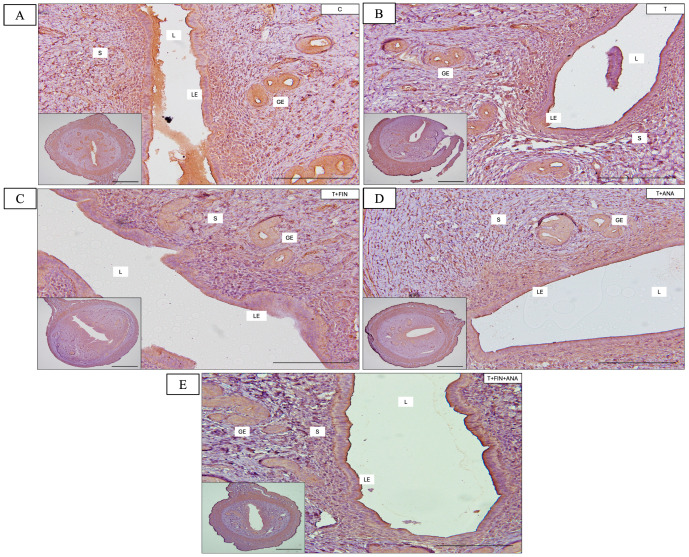

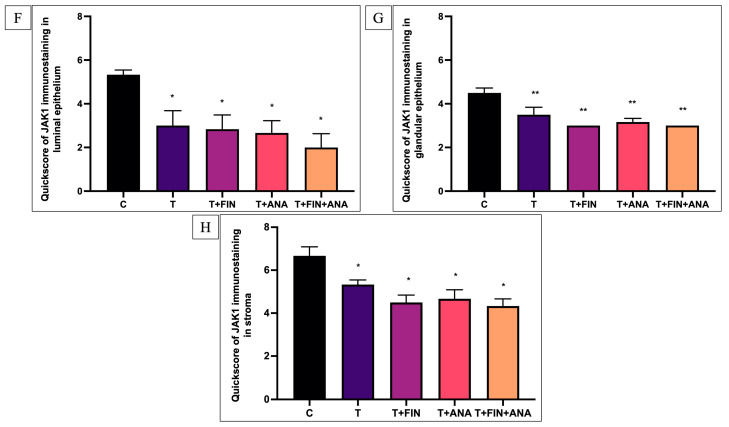
Distribution of Janus kinase 1 (JAK1) in the uterus of rats. (**A**–**E**) The antibody binding site of JAK1, which can be seen in the stroma and luminal and glandular epithelium of the uterus, is coloured brown. Compared to the control group, all four treatment groups (T, T+FIN, T+ANA and T+FIN+ANA) showed a lighter brown discolouration in all compartments. (**F**–**H**) Semi-quantitative assessment of JAK1 immunostaining for the endometrial compartments. LE: Luminal epithelium; GE: Glandular epithelium; L: Lumen of the endometrium; S: Stroma. Scale bar = 200 μm, scale bar insert = 1 mm, magnification 40× and 200×. Error bars represent standard error of the mean (SEM). n = 6 in each group, * *p* < 0.05, ** *p* < 0.01 indicate significance relative to the normal control group. C: control; T: testosterone propionate; T+FIN: testosterone propionate + finasteride; T+ANA: testosterone propionate + anastrozole; T+FIN+ANA: testosterone propionate + finasteride + anastrozole.

Figure 5 illustrates the degree of immunostaining (Quickscore) for JAK1 across various uterine compartments in female rats. In the luminal epithelial compartment, the control group showed the highest Quickscore (~6.5) for JAK1 immunostaining in the luminal epithelium (*p* < 0.05). A comparable trend was noted across all treated groups, namely T, T+FIN, T+ANA and T+FIN+ANA, which exhibited a substantial decrease in JAK1 immunostaining relative to the control group (*p* < 0.05).

As for the glandular epithelium, the control group again showed the highest JAK1 Quickscore. A statistically significant decrease (*p* < 0.01) in JAK1 immunostaining was observed in all treatment groups including T, T+FIN, T+ANA and T+FIN+ANA compared to the control group.

The control group had a higher Quickscore for JAK1 immunostaining in the stroma. Similar to the other uterine compartments, all treatment groups (T, T+FIN, T+ANA and T+FIN+ANA) display a decreased JAK1 distribution, each characterised by a statistically significant reduction (*p* < 0.05) relative to the control group.

**Figure 6:**
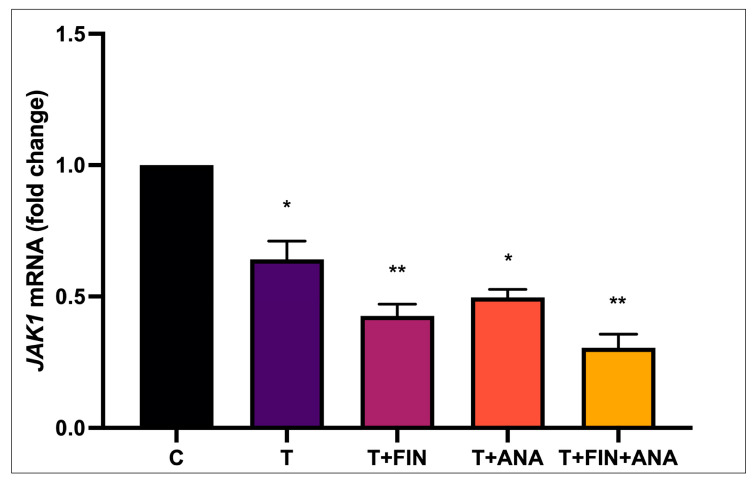
Effects of testosterone on *JAK1* mRNA expression. n = 6 in each group, error bars denote the standard error of the mean (SEM). * *p* < 0.05, ** *p* < 0.01 indicates significance relative to the normal control group. C: normal control; T: testosterone propionate; T+FIN: testosterone propionate + finasteride; T+ANA: testosterone propionate + anastrozole; T+FIN+ANA: testosterone propionate + finasteride + anastrozole.

Figure 6 shows *JAK1* mRNA expression. The control group was set as baseline (1.0). The testosterone group showed a significant downregulation of *JAK1* mRNA (*p* < 0.05). In the cohort receiving the combination of finasteride + testosterone, *JAK1* mRNA was significantly less regulated than in the group with testosterone alone (*p* < 0.01). In the cohort with the combination of anastrozole + testosterone, there was also a partial recovery of *JAK1* expression (*p* < 0.05) relative to the control group. A similar pattern can be seen in the treatment combination of testosterone + finasteride + anastrozole, which demonstrated a more pronounced downregulation (*p* < 0.01) relative to the control group.

### 3.4. Protein Distribution and mRNA Expression of Signal Transducer and Activator of Transcription 3 (STAT3)

**Figure 7:**
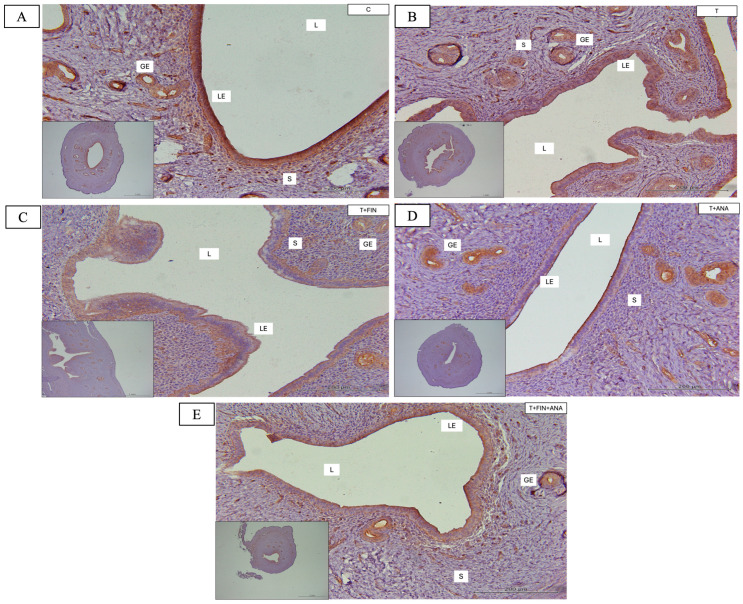

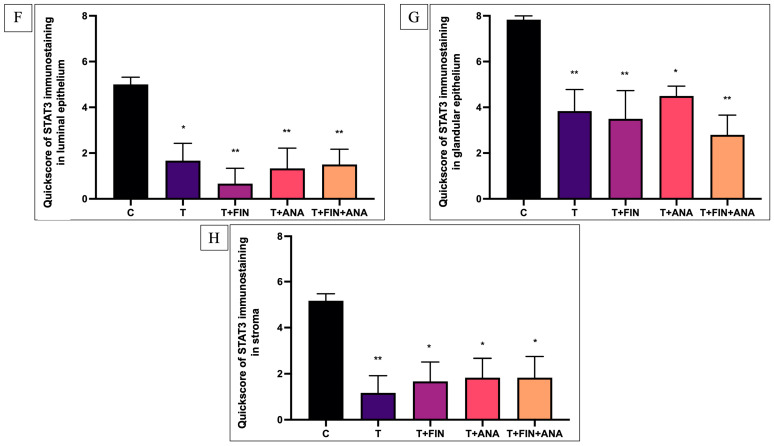
Distribution of signal transducer and activator of transcription 3 (STAT3) in the uterus of rats. (**A**–**E**) The antibody binding site of STAT3, which can be seen in the stroma and luminal and glandular epithelium of the uterus, is coloured brown. Compared to the control group, all four treatment groups (T, T+FIN, T+ANA and T+FIN+ANA) showed a lighter brown discolouration in all compartments. (**F**–**H**) Semi-quantitative assessment of STAT3 immunostaining for the endometrial compartments. LE: Luminal epithelium; GE: Glandular epithelium; L: Lumen of the endometrium; S: Stroma. Scale bar = 200 μm, scale bar insert = 1 mm, magnification 40× and 200×. Error bars denote the standard error of the mean (SEM). n = 6 in each group, * *p* < 0.05, ** *p* < 0.01 indicate significance relative to the normal control group. C: control; T: testosterone propionate; T+FIN: testosterone propionate + finasteride; T+ANA: testosterone propionate + anastrozole; T+FIN+ANA: testosterone propionate + finasteride + anastrozole.

Figure 7 illustrates the distribution and intensity of STAT3 immunostaining within the glandular epithelium, luminal epithelium and stromal compartments of the uterus across five experimental groups. The first graph shows the Quickscore of STAT3 immunostaining in the luminal epithelium. The control group exhibits the highest STAT3 immunostaining value. In contrast, STAT3 immunostaining is significantly reduced in the testosterone group relative to the control group (*p* < 0.05). A significant reduction in STAT3 immunostaining was also observed in the other treatment groups, including testosterone alone or the combination of testosterone with finasteride or anastrozole or both inhibitors, compared to the control group (*p* < 0.01).

As for the glandular epithelium, the control group again showed the highest STAT3 activation. The treatment group with testosterone alone or in combination with finasteride or in combination with both inhibitors showed a significant decrease in STAT3 activity relative to the control group (*p* < 0.01). However, the treatment group receiving testosterone and anastrozole exhibited a slight reduction in STAT3 activity relative to the control group (*p* < 0.05).

In the stromal compartment, the control group had the highest STAT3 activity and displayed a comparable pattern to the other compartments. The testosterone group leads to a substantial decrease in stromal STAT3 activity (*p* < 0.01) compared to the normal control group. In the other treatment groups, testosterone alone or the combination of testosterone with finasteride or anastrozole or both inhibitors, STAT3 activity was also significantly reduced compared to the control group (*p* < 0.05).

**Figure 8:**
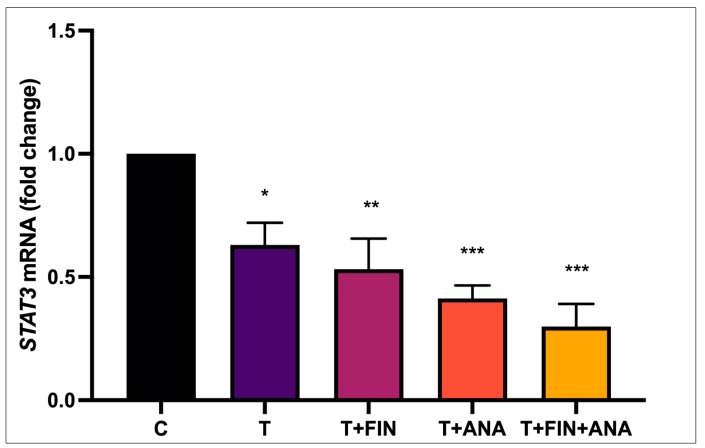
Effect of testosterone on *STAT3* mRNA expression. n = 6 in each group, error bars denote the standard error of the mean (SEM). * *p* < 0.05, ** *p* < 0.01, *** *p* < 0.001 indicate significance relative to the normal control group. C: normal control; T: testosterone propionate; T+FIN: testosterone propionate + finasteride; T+ANA: testosterone propionate + anastrozole; T+FIN+ANA: testosterone propionate + finasteride + anastrozole.

Figure 8 illustrates the expression of *STAT3* mRNA. The control group was defined as the baseline value (1.0). The testosterone group showed a significant downregulation of *STAT3* mRNA (*p* < 0.05). In the testosterone + finasteride group, an even more significant downregulation of *STAT3* mRNA levels was observed compared to testosterone alone (*p* < 0.01). *STAT3* expression decreased substantially in the testosterone + anastrozole group and in the combined therapy of testosterone + anastrozole + finasteride compared to the control group (*p* < 0.001).

### 3.5. Protein Distribution and mRNA Expression of Mucin 1 (MUC1)

**Figure 9:**
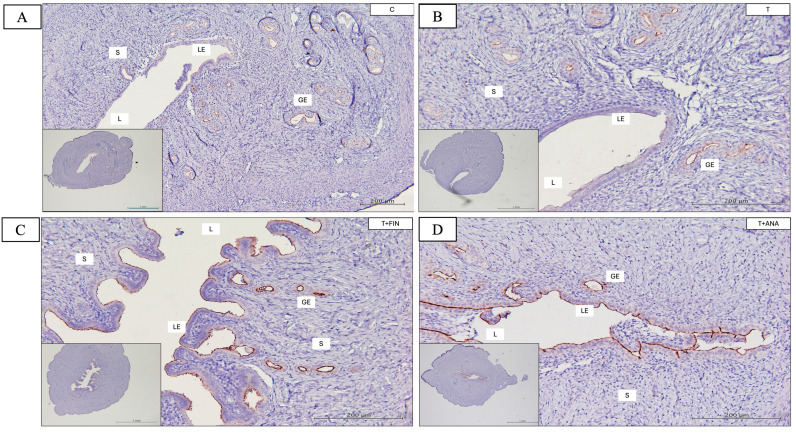

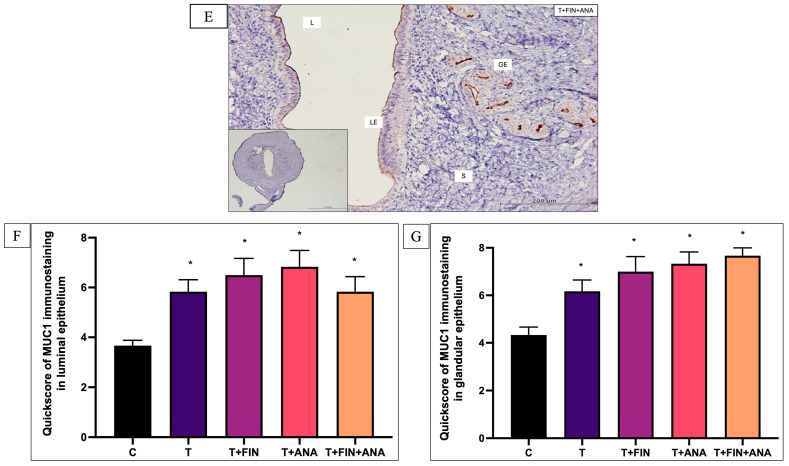
Distribution of mucin 1 (MUC1) in the uterus of rats. (**A**–**E**) The antibody-binding location of MUC1, which can be seen in the stroma and luminal and glandular epithelium of the uterus, is coloured brown. Compared to the control group, all four treatment groups (T, T+FIN, T+ANA and T+FIN+ANA) showed a lighter brown discolouration in all compartments. (**F**,**G**) Semi-quantitative assessment of MUC1 immunostaining for the endometrial compartments., LE: Luminal epithelium; GE: Glandular epithelium; L: Lumen of the endometrium; S: Stroma. Scale bar = 200 μm, scale bar insert = 1 mm, magnification 40× and 200×. Error bars denote the standard error of the mean (SEM). n = 6 in each group, * *p* < 0.05 indicates significance relative to the normal control group. C: control; T: testosterone propionate; T+FIN: testosterone propionate + finasteride; T+ANA: testosterone propionate + anastrozole; T+FIN+ANA: testosterone propionate + finasteride + anastrozole.

Figure 9 shows the Quickscore of the immunostaining for the distribution of MUC1 in the glandular epithelium and in the luminal epithelium of the rat uterus in five test groups. The control group in the luminal epithelium had the lowest Quickscore, indicating the MUC1 expression at baseline. In contrast, the Quickscore was slightly increased in the testosterone group, which was deemed statistically significant (*p* < 0.05). A similar pattern can be seen in the other treatment groups such as testosterone alone or the combination of testosterone with finasteride or anastrozole or both inhibitors, which showed a significant increase (*p* < 0.05) in MUC1 immunostaining compared to the control group.

In the glandular epithelium, MUC1 immunostaining activity showed the lowest Quickscore in the control group compared to the treatment groups. Similar to the luminal epithelium, the Quickscore showed a significant increase (*p* < 0.05) in all treatment groups compared to the control group.

**Figure 10:**
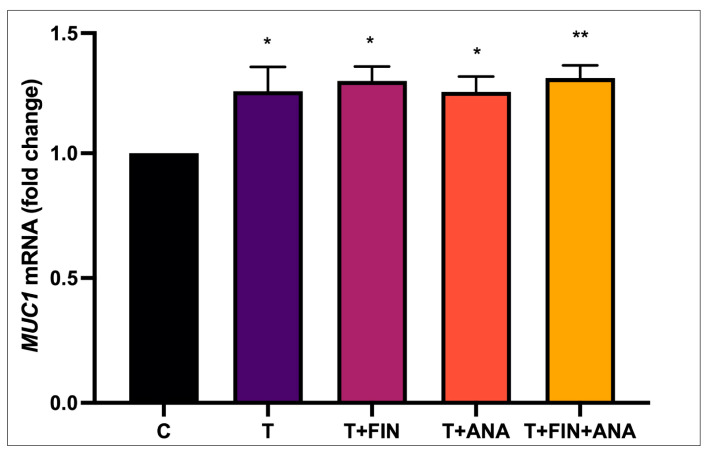
Effect of testosterone on *MUC1* mRNA expression. n = 6 in each group, error bars denote the standard error of the mean (SEM)., * *p* < 0.05, ** *p* < 0.01 are significant compared to the normal control group. C: normal control; T: testosterone propionate; T+FIN: testosterone propionate + finasteride; T+ANA: testosterone propionate + anastrozole; T+FIN+ANA: testosterone propionate + finasteride + anastrozole.

Figure 10 shows the *MUC1* mRNA expression. The control group was set as baseline (1.0). The group with testosterone alone (T) and with the addition of the inhibitors (T+FIN and T+ANA) showed a significant upregulation of *MUC1* mRNA (*p* < 0.05). The group with the treatment combination of testosterone + anastrozole + finasteride (T+FIN+ANA) showed a further upregulation of *MUC1* expression (*p* < 0.01) compared to the control group.

## 4. Discussion

The leukaemia inhibitory factor (LIF) signalling pathway plays a central role in the regulation of endometrial receptivity during the implantation window. This signalling pathway is crucial for mediating cellular communication, differentiation and signalling between the embryo and the endometrium [26]. In this study, we investigated the effect of a supraphysiological dose of testosterone on the LIF-JAK1-STAT3 signalling pathway, providing important insights into how excessive androgen exposure might disrupt this signalling environment, leading to reduced endometrial receptivity and infertility.

The influence of testosterone, one of the most important androgens, on female reproductive function is increasingly recognised. However, supraphysiological testosterone levels can disrupt the finely tuned hormonal balance that is crucial for endometrial receptivity [27]. In this study, it was observed that the administration of a supraphysiological dose of testosterone (1 mg/kg/day) significantly decreased the mRNA expression and protein distribution of LIFR, JAK1 and STAT3 in the endometrium, affecting both the luminal and glandular epithelium, as well as the stromal compartments. The combination of testosterone with finasteride, an androgen receptor (AR) inhibitor, and anastrozole, an aromatase inhibitor, provided further insights into the pathways by which testosterone exerts its effect.

This study shows a significant decrease in both *LIF* and *LIFR* mRNA expression and decreased LIFR distribution in all endometrial compartments (luminal epithelium, glandular epithelium and stroma) in all treatment groups. This suggests that elevated testosterone impairs the signalling initiation required for downstream activation of the JAK1-STAT3 pathway during the receptive phase. Savaris et al. (2011) revealed that the expression of LIF in the endometrium of women with polycystic ovary syndrome (PCOS) was significantly lower in the mid-secretory phase than in normal women, suggesting poor endometrial receptivity in PCOS patients [28]. Other studies have also reported that LIF-deficient female mice have an implantation defect that can be corrected by supplementation with LIF [29,30].

In addition, LIF is known to be influenced by hormonal regulation, particularly oestrogen and progesterone, during the oestrous cycle. However, excess testosterone can interfere with this regulation, possibly through androgen receptor (AR) signalling, which can suppress oestrogen-mediated signalling pathways and disrupt LIF expression. This interference is consistent with research showing that androgen excess can alter normal endocrine signalling and impair endometrial receptivity [31]. This downregulation is consistent with the study by Si et al. (2024) showing that women with high total testosterone levels had significantly lower clinical and sustained pregnancy rates and that the expression of LIF and insulin-like growth factor binding protein 1 (IGFBP-1) in the endometrium also decreased significantly. In addition, high doses of testosterone significantly downregulated the expression of IGFBP-1 and LIF in Ishikawa cells [31].

On the other hand, our results showed that the expression of *JAK1* mRNA and *STAT3* mRNA was also significantly reduced, and the protein distributions of both were decreased in all endometrial compartments compared to the control group. This indicates a downstream suppression of the LIF-JAK1-STAT3 signalling pathway due to reduced LIF signalling. Normally, the activation of LIFR and gp130 leads to the initiation of cytoplasmic JAK [32] and STAT 1/3 [33]. However, the significant reduction in both *JAK1* mRNA and *STAT3* mRNA and protein distribution in all testosterone-treated groups underlines the disruption of this signalling pathway. In support of our data, a study by Yen et al. (2017) reported that significantly reduced LIFR expression and subsequent phosphorylation of STAT3 and extracellular signal-regulated kinase (ERK) in the eutopic endometrium was found in patients with adenomyosis [13]. The expression of STAT3 is closely associated with successful implantation. The phosphorylation of STAT3 and subsequent translocation to the nucleus is essential for the transcription of pro-receptive genes. The observed decrease in STAT3 expression in all endometrial compartments indicates that high-dose testosterone strongly impairs the endometrial environment necessary for implantation. The activation of STAT3 is essential for the regulation of genes involved in cellular adhesion and remodelling during the implantation window [34]. Reduced STAT3 activity could lead to impaired decidualisation and a hostile environment for embryo implantation [13]. This is supported by research showing that impaired STAT3 signalling is associated with infertility or poor pregnancy outcomes [7]. As evidence, it has been shown in a conditional knockout mouse model that the activation of STAT3 in glandular cells is responsible for disrupting cell–cell contact of the endometrial luminal epithelium to promote the implantation process [34]. Therefore, the significant reduction in STAT3 activation might not break the contacts between endometrial cells and hinder the process of embryo implantation [13].

Interestingly, in our study, an increase in *MUC1* mRNA expression and protein distribution was observed in all endometrial compartments, despite reduced LIF-JAK1-STAT3 signalling. MUC1 is a transmembrane glycoprotein that normally prevents embryo attachment by forming a barrier. MUC1 is highly expressed in the uterine epithelium on day 1 of pregnancy, and its expression is downregulated in the epithelium when endometrial receptivity is reached on day 4 [35]. The upregulation of MUC1 in response to high-dose testosterone suggests a shift towards a non-receptive state of the endometrium. It is possible that reduced LIF-STAT3 signalling did not sufficiently suppress MUC1 expression, resulting in excessive mucin production. This is consistent with studies showing that the inadequate regulation of MUC1 is associated with lower implantation success [13]. The expression of MUC1, which was highest at the apical membrane of the uterine luminal epithelium, also supports its role in preventing adhesion between the blastocyst and the endometrium [36]. In contrast to these recent findings, a study by Mokhtar et al. (2018) reported that the expression of MUC1 was downregulated in a state of androgen excess during the receptive period [37]. Under both conditions, i.e., both the upregulation and downregulation of MUC1, embryo implantation is therefore highly likely to fail.

In addition, testosterone significantly reduces the thickness of the endometrium and myometrium, as well as the number of glands, which indicates a disruption of the uterine architecture. The additional administration of finasteride or anastrozole alone does not appear to completely counteract the effects of testosterone on these parameters. The combined treatment (T+FIN+ANA) resulted in the most profound reductions in all parameters, suggesting a stronger inhibitory effect when both the 5α-reductase and aromatase pathways are blocked together with testosterone. These data suggest that both the androgenic and oestrogenic pathways are involved in the maintenance of the uterine structure and that disruption of both pathways can significantly alter tissue morphology. However, there are studies that report that with prolonged administration of testosterone, endometrium histological analysis showed a significant increase in luminal epithelial height and glandular density without changes in cell proliferation. The thickness of the subepithelial stroma and the myometrium also increased in these rats [38,39].

Meanwhile, the findings of the combination groups, including testosterone plus finasteride and testosterone plus anastrozole, shed light on the effects of the pathway on testosterone. Finasteride is a 5α-reductase inhibitor that blocks the conversion of testosterone to dihydrotestosterone (DHT), the more potent androgen. However, despite blocking DHT formation, the suppression of LIF-JAK1-STAT3 signalling persisted in this group. This suggests that the deleterious effects of testosterone may occur via both DHT-independent mechanisms and AR-mediated pathways directly through testosterone, as AR signalling may continue to impair LIF expression [40].

In addition, anastrozole is an aromatase inhibitor that prevents the conversion of testosterone to oestrogen. The reduction in oestrogen levels may have contributed to the decreased LIF expression in this group, as oestrogen is necessary for the upregulation of LIF during the peri-implantation period [30]. This is consistent with the findings that oestrogen deficiency can impair implantation by lowering LIF and other cytokine levels [41]. The combined effects of androgen excess and oestrogen deficiency likely suppress LIF production and disrupt the receptivity signalling cascade, suggesting that both hormonal imbalances contribute to impaired implantation potential.

## 5. Conclusions

In conclusion, this study suggests that supraphysiological doses of testosterone, even when modulated by inhibitors such as finasteride and anastrozole, lead to significant perturbations of the LIF-JAK1-STAT3 pathway. The decreased expression of LIF, LIFR, JAK1 and STAT3 in the endometrial compartments suggests that androgen excess disrupts the molecular mechanisms required for endometrial receptivity. The increase in MUC1 expression emphasises the shift towards a non-receptive endometrium, which is likely to prevent successful embryo implantation. This disruption of critical signalling pathways highlights the need for further investigation of androgenic effects on endometrial receptivity, particularly in the context of fertility disorders associated with hyperandrogenism. Understanding the molecular basis of the effects of testosterone on implantation will be critical for the development of targeted therapies to restore endometrial function and improve reproductive outcomes.

## Figures and Tables

**Table 1:** Quickscore assessment.

Proportion Score	% of Positive Cells	Intensity	Intensity Score
**0**	0	None	0
**1**	<1	Weak	1
**2**	1 to 10	Intermediate	2
**3**	11 to 33	Strong	3
**4**	34 to 66	QS (intensity score + proportion score): 0–8	
**5**	>67		

## Data Availability

The original contributions presented in the study are included in the article, and further inquiries can be directed to the corresponding author.
